# Editorial: Uterine factors associated with fertility impairment

**DOI:** 10.3389/fendo.2023.1307237

**Published:** 2023-11-07

**Authors:** Lusine Aghajanova, Signe Altmäe, Anna Sokalska

**Affiliations:** ^1^ Division of Reproductive Endocrinology and Infertility, Department of Obstetrics and Gynecology, Stanford University School of Medicine, Stanford, CA, United States; ^2^ Department of Biochemistry and Molecular Biology, Faculty of Sciences, University of Granada, Granada, Spain; ^3^ Instituto de Investigación Biosanitaria ibs.GRANADA, Granada, Spain; ^4^ Division of Obstetrics and Gynecology, Department of Clinical Science, Intervention and Technology (CLINTEC), Karolinska Institutet and Karolinska University Hospital, Stockholm, Sweden

**Keywords:** uterus, endometrium, implantation failure, adenomyosis, endometriosis, chronic endometritis, endocrine disruptors, microbiome

Infertilty is a prevalent medical condition affecting 9-18% of the global population with substantial impact on both the individual and societal level. While there have been noteworthy advancements in understanding the etiology, progression, and treatment of male and female factor infertilty, challenges remain for the medical community. Even at cutting-edge institutions, the success rate for treating infertility plateaus around 60%. We posit that augmented research efforts can help to address more complex cases and push the success rate further.

In particular, the uterine environment and endometrial health are essential contributors to fertility outcomes. The goal of this Special Reproduction Research Topic of Frontiers in Endocrinology is to address current challenges related to uterine and endometrial physiology and pathology and to highlight novel basic, translational, and clinical research in the field. We specifically seek to update readers on developments in endometriosis, adenomyosis, endometrial receptivity, recurrent implantation failure, management of uterine septum, pathophysiology of endometritis, endometrial microbiome, and endocrine disruptors’ effect on endometrial function.

To begin, we want to draw readers’ attention to a comprehesive review on endometriosis by Bonavina and Taylor. This article addresses everything you want to know about endometriosis, it covers pathophysiology, genetics and epigenetics, mechanisms of infertility, clinical presentation, and association with adenomyosis and other uterine issues. The authors then summarize the available treatment modalities including medical, surgical, and emerging therapies. This review provides a strong background for a nuanced translational study completed by Huang et al. addressing the potential use of human adipose-derived stem cell conditioned medium to improve endometriosis burden and subsequnt fertility in a murine model of endometriosis. The success of this therapy is likely attributed to its anti-inflammatory and antiangiogenic properties. Readers should stay tuned for possible implementation of this strategy in humans using Good Tissue Practice and Good Manufacturing Practice regulations. Keeping in mind the multifaceted nature of endometriosis and its rare but potential transformation to malignancy, we want to highlight a study by Collins et al. The authors aimed to explore the molecular mechanisms behind the transformation of ovarian endometriosis (as a model for cystic lesions of deep endometriosis) to ovarian clear-cell carcinoma. Through deep transcriptomic and correlation analysis, they discovered dysregulation at the level of mRNA and miRNA, which was also confirmed during *in vitro* experiments. The authors eventually suggested miR-10a-5p as a molecule with oncogenic potential worth further evaluation.

Moving from endometriosis to adenomyosis, a similar but somewhat different condition, we would like to guide readers towards a paper by He et al. investigating a potential mechanism for impared endometrial receptivity in patients with adenomyosis. Elegantly using human endometrial cells *in vitro* and an adenomyosis mouse model *in vivo*, the authors showed that IL-33 is dysregulated in adenomyosis and linked to endometrial HOXA10 function. The reduced expression of IL-33 in the endometrium in adenomyosis impairs embryo implantation through modulation of HOXA10 expression through the STAT3 pathway. Tang et al. focused thei research efforts on understanding the mechanism of action of the dopamine receptor agonist bromocriptine as a novel medical management option for adenomyosis. Their publication reported that bromocriptine treatment exhibited an anti-proliferative effect within the endometrium of patients with adenomyosis *in vivo* and *in vitro*. This effect could be related to subsequent upregulation of several miRNAs and is consistent with symptomatic improvement in adenomyosis patients after bromocriptine therapy.

As the ultimate goal of infertility management is achieving a live birth, Wu et al. attempted to create a model to predict the chance of live birth after frozen embryo transfer (FET) in patients with adenomyosis, which was later validated in different sets of patients. The authors built a statistical nomogram incorporating age, uterine volume, developmental stage of the transferred embryo, FET protocol, and presence of singleton versus twin pregnancy after selecting from numerous variables. The results showed that younger age, smaller uterine volume, blastocyst transfer, and hormone replacement FET protocol with GnRH-agonist were associated with better live birth outcomes, while achieving a twin pregnancy was associated with a lower chance of live birth.

Switching gears to anatomic issues, we would like to update readers on the highly-debated management of a septate uterus. Chang et al. retrospectively analyzed a large database from a single institution where uterine septum resection was performed on all patients with infertility, miscarriage, or poor obsteric outcomes, regardless of prior pregnancy outcomes. The authors reported that live birth, clinical pregnancy, and early miscarriage rates were all significantly improved after surgery in patients with a septate uterus and maternal age ≥ 35 years. Primary infertility was associated with a lower chance of clinical pregnancy after septum resection. Although the results of this retrospective analysis contrast those of the only completed randomized controlled trial on this topic (1), certain methodological and clinical factors might have influenced the variation in outcomes.

Another controversial topic is recurrent implantation failure (RIF), a clinical phenomenon characterized by lack of implantation after at least 3 euploid blastocyst transfers (or the equivalent number of unscreened embryo transfers adjusted to the patient’s age and corresponding euploidy rate). While true RIF arguably is extremely uncommon, occuring in less than 5% of couples with infertility (2), the nature of this reproductive impairment is not clearly established. One of the putative causes underlying RIF are alterations in endometrial receptivity. Several recent studies showed that “personalized” embryo transfer based on Endometrial Receptivity Assay (ERA) for an unselected group of patients (all comers) did not improve pregnancy outcomes compared to conventional transfer timing. In an effort to idenfity an appropriate test for the implantation window of patients with RIF, Chen et. al. conducted a study comparing an RNA-sequencing-based endometrial receptivity test (rsERT) with pinopode evaluation. After personalized embryo transfer, patients in rsERT group had significantly higher pregnancy rates and required fewer FET cycles, supporting the use of rsERT as a potential clinical tool. Other potential causes of RIF are immunological abnormalities. Guo et al. studied the alterations of cytokine profiles in patients with RIF, trying to shed new light on its pathomechanism. Several elevated proinflammatory cytokines, decreased anti-inflammatory cytokines, and an increased Th1/Th2 cytokine ratio were observed in the mid-luteal serum of RIF patients compared to control subjects. A constructed predictive model for RIF found that IL-10, G-CSF and IL-2/IL-10, in particular, had independent predictive values for occurrence of RIF. Furthermore, in combination with age, and endometrial thickness, IL-6 and IFN-y/IL-10 demonstrated good diagnostic performance.

Various therapeutic options have been proposed to manage RIF without groundbreaking success. A study by Niu et al. aimed to explore the effects of low molecular-weight heparin (LMWH) treatment on the uterine inflammatory cytokine profile and pregnancy outcomes of patients with RIF but without trombophilia. The cytokine mRNA profile in endometrial samples obtained from 326 RIF patients undergoing FET with or without LMWH treatment demontrated different patterns potentially suggesting beneficial action of LMWH on implantation. Although the clinical and ongoing pregnancy rates did not significantly differ between the two groups, the cohort of patients treated with LMWH and who successfully concieved had significantly higher levels of IL-6, IL-15 and G-CSF. Another approach to improve success in patients with implantation failures was examined by Wang et. al. Several prior studies explored the effects of endometrial injury on clinical pregnancy outcomes with conflicting results. Wang et al. focused their prospective cohort study on the timing of endometrial scratch biopsy. In patients with a history of ≥ 2 failed transfers, the implantation and clinical pregnancy rates were significantly higher if endometrial injury took place in the luteal phase of the cycle preceding embryo transfer (7 days after ovulation), than in follicular phase of the embryo transfer cycle (cycle day 3-5). Interestingly, the endometrial injury did not improve the pregnancy outcomes in unselected patients.

Chronic endometritis (CE) is another condition potentially affecting endometrial receptivity and associated with poor *in vitro* fertilization (IVF)/FET outcomes. The mechanisms underlying CE are not fully established, while unfavorable uterine microenvironment has been suggested as a potential cause. In the search of possible mechanisms, a study by Liu et al. investigated the hypoxic microenvironment and endometrial vascularization in the receptive phase endometrium from infertile women with CE. Their study found that upregulation of the hypoxia factor (H1F1α) and angiogenic factors, including VEGFA and VEGFR2, as well as excessive vascularization in the peri-implantation endometrium, seem to reduce endometrial receptivity in infertile patients with CE. Interestingly, the authors repeated the analyses after first-line antibiotic treatment, and suggested that antibiotic-mediated remodeling of hypoxic and angiogenic homeostasis in the endometrium might improve reproductive outcomes. Nevertheless, these initial results should be subject to further testing and confirmation in future studies.

A thought-provoking study by Molina et al. evaluated the influence of a Mediterranean diet on endometrial whole metabolome profile in the receptive phase. The study provided insight into the molecular background of “endometrial well-being” and, importantly, the possibilities for modulating endometrial environment and enhancing embryo implantation through nutrition.

In line with the uterine microenvironment, there is notable interest in detecting and identifying the endometrial core microbial composition, termed “microbiota,” in physiology and pathophysiology. A study by Canha-Gouveia et al. assessed the female upper reproductive tract microbial composition in disease-free tissue and found that the endometrium and fallopian tubes share 70% of the bacteria, while the rest is organ-specific. They also showed that the endometrial sampling method may influence the results as well as the fact that each individual possesses their unique microbial profile. This knowledge adds to the appreciation of the vast complexity of the natural microenvironment of the female reproductive tract and further understanding with regard to implications on improving IVF/FET protocols.

Finally, we will shift our focus to the external environment. Increasing evidence suggests that environmental pollution has a direct impact on our health. Chemicals that can interfere with the endocrine system are termed endocrine disrupting chemicals (EDCs). Multiple studies have found associations between exposure to EDCs and reduced fertility in women (3). However, there is little knowledge of the effect of EDCs on the endometrium and its function. In a study by Lavogina et al. the effect of a wide selection of EDCs on primary human endometrial stromal cell (hESC) decidualization was evaluated *in vitro.* The authors showed that EDCs commonly present in the blood can reduce decidualization of hESCs. The effects of EDCs on endometrial dysfunction and risk of implantation failure are the next targets of study.

Although we have made significant advancements in understanding embryonic development and selection criteria, our comprehension of uterine and endometrial functions, as well as their associate anomalies, remains insufficient. We hope that this Special Reproduction Research Topic provides more insight into the uterine factors of infertility, stimulates readers’ minds, and provokes new ideas for future research directions.

## Author contributions

LA: Writing – original draft, Writing – review & editing. SA: Writing – original draft, Writing – review & editing. AS: Writing – original draft, Writing – review & editing.

## References

[1] Rikken JFW Kowalik CR Emanuel MH Bongers MY Spinder T Jansen FW. Septum resection versus expectant management in women with a septate uterus: an international multicentre open-label randomized controlled trial. Hum Reprod (2021) 36:1260–7. doi: 10.1093/humrep/deab037 PMC8058590 33793794

[2] Pirtea P Cedars MI Devine K Ata B Franasiak J Racowsky C. Recurrent implantation failure: reality or a statistical mirage?: Consensus statement from the July 1, 2022 Lugano Workshop on recurrent implantation failure. Fertil Steril (2023) 120:45–59. doi: 10.1016/j.fertnstert.2023.02.014 36822566

[3] Silva ABP Carreiró F Ramos F Sanches-Silva A. The role of endocrine disruptors in female infertility. Mol Biol Rep (2023) 50:7069–88. doi: 10.1007/s11033-023-08583-2 PMC10374778 37402067

